# Facilitating small group learning in the health professions

**DOI:** 10.1186/s12909-020-02282-3

**Published:** 2020-12-03

**Authors:** Annette Burgess, Christie van Diggele, Chris Roberts, Craig Mellis

**Affiliations:** 1 The University of Sydney, Faculty of Medicine and Health, Sydney Medical School - Education Office, The University of Sydney, Sydney, NSW 2006 Australia; 2 The University of Sydney, Faculty of Medicine and Health, Sydney Health Professional Education Research Network, The University of Sydney, Sydney, Australia; 3 The University of Sydney, Faculty of Medicine and Health, The University of Sydney, Sydney, Australia; 4 The University of Sydney, Faculty of Medicine and Health, Sydney Medical School, Central Clinical School, The University of Sydney, Sydney, Australia

**Keywords:** Small group learning, Team building skills, Facilitation, Peer assisted learning, Health professional students

## Abstract

There is now good evidence that small group teaching provides a fruitful academic environment, which optimises learning, particularly in the healthcare setting, and especially when compared to lectures. An individual student’s understanding of knowledge is increased when they are able to actively compare and build on their own understanding in conjunction with their peers. Small group teaching provides opportunities for learners to work collaboratively, and promotes team-building skills – skills that are essential to work within healthcare settings. The aim of this paper is to provide health professional students and early career health professionals involved in peer and near peer teaching, with an overview of approaches and tips to improve learner engagement when facilitating small groups.

## Background

Health professional education occurs in a variety of contexts, including those within university, hospital, community-based and clinical settings. Curricula activities at the university target development of students’ knowledge of the basic sciences of healthcare (such as physiology, pathology, and anatomy), which are then integrated into the clinical setting, thus contextualising this knowledge. The clinical setting also plays a crucial role in developing students’ clinical skills, communication skills, and professionalism. The clinical application of the basic sciences also occurs in case scenario based small group teaching methods, such as problem based learning (PBL), Team-based learning (TBL), Case based learning (CBL), in the university setting [1–7]; and communication skills, clinical skills, and procedural skills teaching in the clinical, patient-based setting [8, 9]. Compared to lecture based teaching, these small group methods provide a more fruitful academic environment, and maximise student learning [10], and remain the preferred approach to pedagogy in health professional education [5].

An individual student’s understanding of knowledge is increased when they are able to actively compare and build on their own understanding in conjunction with their peers [11–16]. Small group teaching provides opportunities for learners to work collaboratively with their peers, and promotes team building skills – skills that are essential to working within healthcare settings [17, 18]. However, all learning experiences are only as effective as the students’ engagement with them. While some small group learning experiences are inviting and supportive, others may impede efforts to learn [19]. The aim of this paper is to provide health professional students and early career health professionals involved in peer and near peer teaching, with an overview of approaches and tips to improve learner engagement when facilitating small groups.

### What defines small group learning, and what are the benefits?

The suggested ideal group size for small group learning is between five and eight people, with six often being considered ‘optimal’ [20, 21]. However, small group learning is not defined by the number of participants, and should not be confused with a lecture to small groups. In order for the lesson to be classed as a small group learning experience it must involve three key elements [22]: *active participation, ‘face-to-face’ contact between participants,* and *purposeful activities*. When implemented with all three elements in play, the small-group context offers many benefits, and enhances students’ learning experiences in many ways. For example, small group learning has the potential to: [15, 16, 21, 23]

- Help address gaps in student knowledge
- Encourage self-directed learning
- Allow students to engage with a range of perspectives from their peers
- Allow students to test their ideas and attitudes with their peers
- Promote a willingness for students to share their ideas
- Provide opportunities for students to give and receive feedback
- Help students to develop skills in critical thinking and problem solving
- Help students to develop communication, teamwork and leadership skills.

### What is the role of the facilitator in small group teaching?

**Fig. 1:**
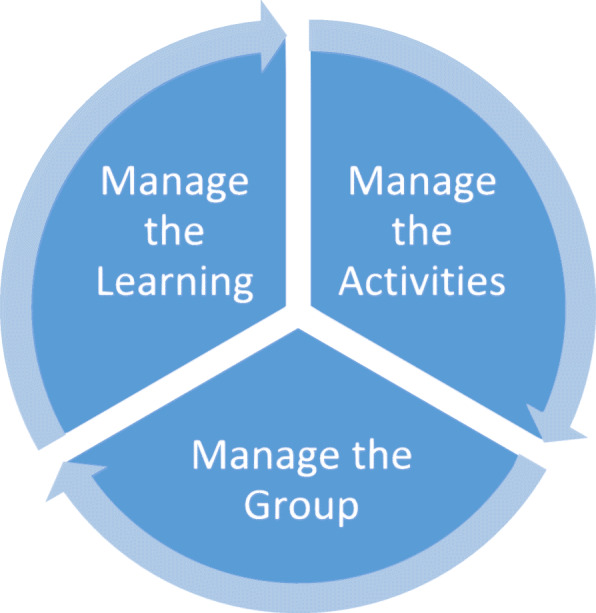
Three key roles of the facilitator in small groups

Rather than being facilitator centred, small group teaching is designed to be learner focused [24]. Actively involving students in learning leads to increased interest, teamwork ability, improvement in self-directed learning and better retention of knowledge and skills [23, 24]. There are three main happenings (Fig. 1) that small group facilitators must manage simultaneously: the group, the activities and the learning [25, 26].

The role of the facilitator is to ‘facilitate’ the learning: lead the discussion, ask open-ended questions, guide the process and ensure active participation from students [26]. However, a range of roles may need to be adopted in order to respond to the way small groups function and interact. During a small group teaching session, the facilitator’s specific roles include:

- Setting clear goals at the start of the session
- Facilitating the session and ensuring it runs on time
- Maintaining the flow of content, ensuring a logical sequence of learning, and provision of stimulating material and questions
- Questioning students to check their understanding
- Encouraging students to ask questions throughout the session
- Clarifying areas that may cause misunderstanding or confusion for students
- Providing effective feedback
- Managing the group dynamics, including resolving conflict and unprofessional behaviour
- Critical reflection and lesson evaluation at the conclusion of the teaching session.

### Small group interaction

There is a need to understand the internal dynamics of the group and how to manage different learners. Tuckman’s (1965) framework provides a useful way of thinking about the ways in which group dynamics develop over time [26, 27]. According to Tuckman, there are five key phases in small group team development:

1. *Forming* - the initial formation of a group. Facilitators are responsible for facilitating introductions, implementing ice-breaker tasks, explaining the activities and purpose of the group.
2. *Norming* - ideas are shared within the group and rules are developed. The facilitator is responsible for encouraging everyone to participate, clarifying ground rules, and ideas/suggestions the group may have regarding the process.
3. *Storming* - the group actively tries to perform the task, however some conflict may arise within the group. The facilitator assists by moderating conflicts and clarifying ideas.
4. *Performing* - the group starts to form a team approach to performing the set tasks. The facilitator keeps the group focussed.
5. *Closure* – includes ‘adjourning’ after each session, or, ‘mourning’, when a group has successfully worked together, completed their tasks and dissolves (the final stage).

### Strategies to consider as a facilitator

Initially, an appropriate culture needs to be established within the group [26]. In getting started, the facilitator should contribute to the creation of a positive and comfortable learning environment by:

- Ensuring the room is set up appropriately (consider seating, noise, privacy)
- Planning effective introductions (‘ice breakers’)
- Outlining expectations and ground rules (e.g. maintaining confidentiality)
- Learning and using the students’ names
- Discussing and assigning roles and responsibilities to group members (eg. timekeeper; scribe)
- Determining the learning needs of individuals by asking questions or observing performance, and gaining and understanding of the learner’s level of knowledge and skills.

**Fig. 2:**
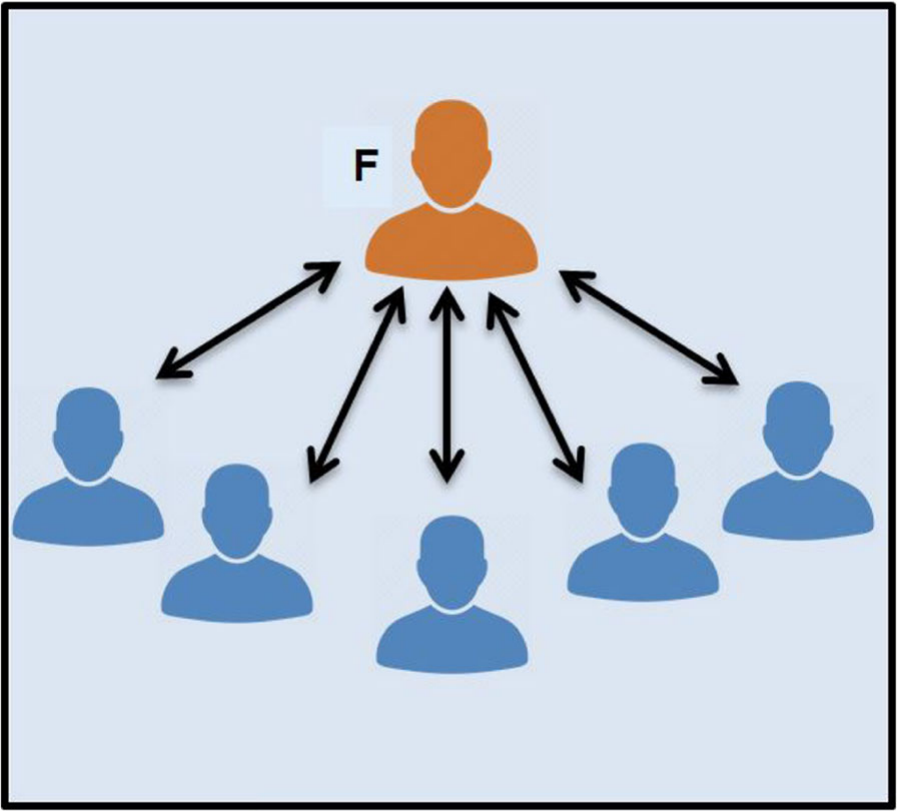
Didactic interactions between the facilitator (F) and individual learners. (Adapted from McKimm & Morris, 2009) [26]

**Fig. 3:**
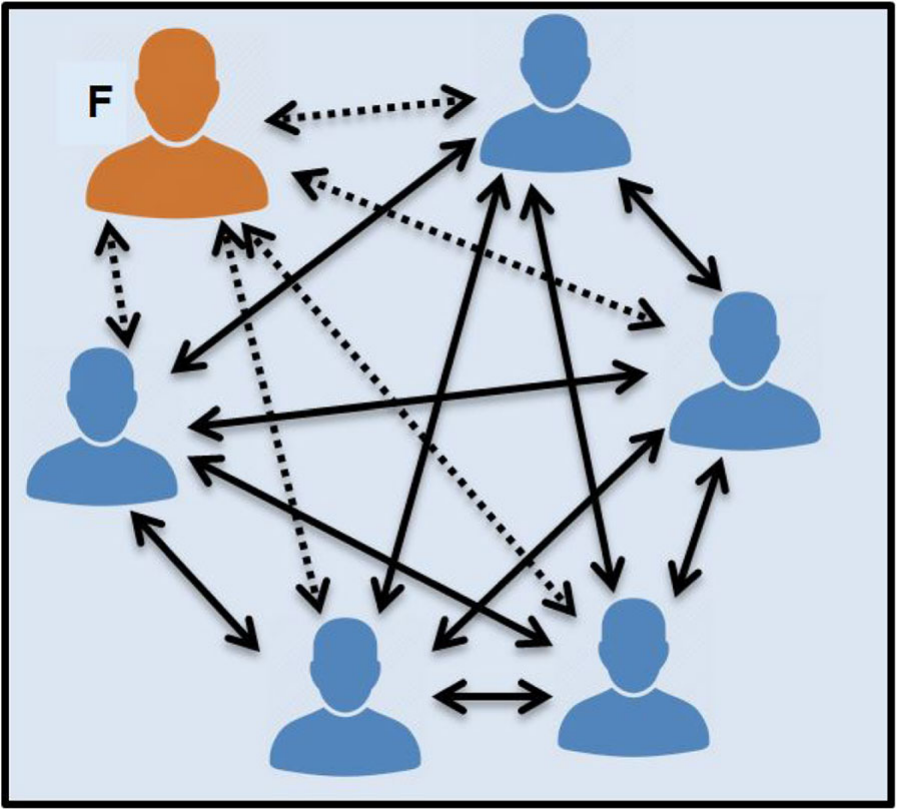
Multiple, active interaction between the facilitator (F), individual learners and their peers. (Adapted from McKimm & Morris, 2009) [26]

Strategies that foster interactions between learners include: buzz groups, where students are given an opportunity to discuss a topic for a specific amount of time; role play/simulation; creation of a poster/drawing; and break out activities [26]. As demonstrated by comparing Fig. 2 with Fig. 3, using different forms of questioning to shift the students’ focus helps to facilitate discussion and promote interaction [26, 28]. In Fig. 2, the small group is not working. This is a ‘lecture’ with no interaction between learners. In Fig. 3, the small group is working well, with lots of interaction between learners and the facilitator, but the facilitator does not have a dominant role (i.e. they are facilitating).

### Questioning in small group teaching

The use of frequent questions in small group facilitation helps to create a learner-centred approach to learning. This allows the facilitator to gain an understanding of the learning needs of individual students, enabling them to pitch their response and interactions at an appropriate level [29]. The use of questioning promotes clinical reasoning, encourages reflection, and enables the facilitator to monitor the learners’ progress. For example, the use of closed questions requires only a single answer, while the use of open questions requires the learner to combine pieces of information and formulate an answer [30, 31]. Different questioning strategies promote different responses, stimulating deeper thinking, reflection and discussion. Some examples of questioning strategies include:

- **Evidence:** ‘What evidence is there to support that?’
- **Clarification:** ‘Can you explain what that means?’
- **Explanation:** ‘Why do you think that would be the case?’
- **Linking:** ‘How does this idea support what we mentioned earlier on?’
- **Hypothetical:** ‘What would happen if?’
- **Summary and synthesis:** ‘What are we still uncertain about?’

**Fig. 4:**
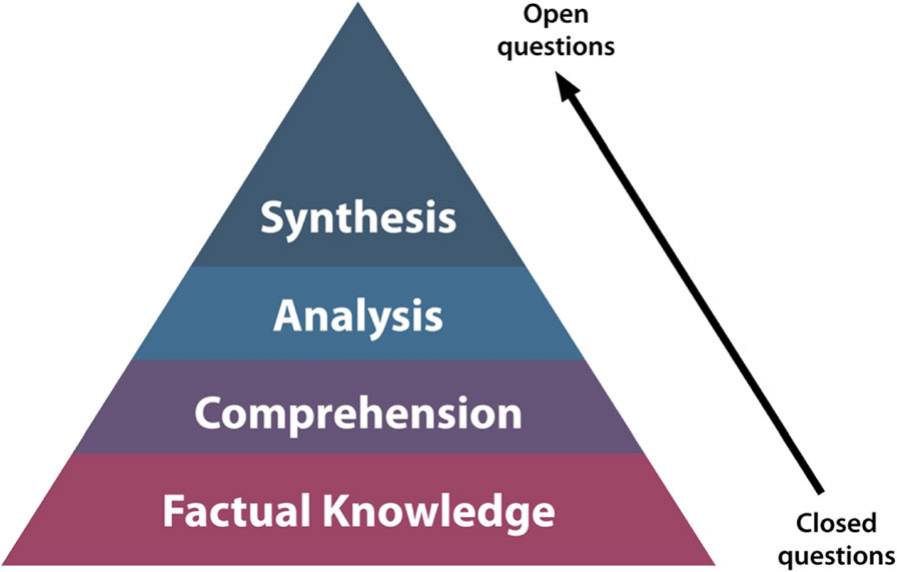
The use of open questions promotes synthesis of information

The appropriate use of questions has the capacity to arouse curiosity, and encourage critical thinking [31]. Importantly, questions can assist the facilitator in assessing the extent of students’ knowledge, and also helps the student to identify their own knowledge gaps. Figure 4 suggests how the use of questions can help promote synthesis of information.

Generally, there are three types of questions [31]:

1. *Yes/No questions:* basic form of questioning, very simple and does not stretch the learner.
2. *Closed questions:* there is a specific answer, enabling the questioner to check the knowledge of the learner, but not their level of understanding.
3. *Open questions:* there is generally no ‘right’ answer. Allows the questioner to probe further asking ‘why’ and ‘how’ type questions. This requires a good understanding of the topic, thinking skills, and problem solving.

**Fig. 5:**
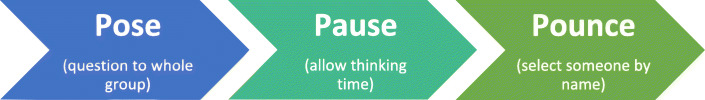
The 3 Ps of questioning (adapted from Lake, Vickery, Ryan, 2005) [31]

In some circumstances, it can be useful to employ the technique of “Pose; Pause; Pounce” (Fig. 5) [31]. After questioning a learner, it is important to pause to allow the learner to register what you are asking, and to think about their response. It is important to allow for this silence and not jump in to rephrase the question immediately, or answer the question yourself [31].

### Facilitator reflection

Critical reflection is considered an essential step to effective education in healthcare, and should be practiced by facilitators [31]. This involves thinking about the facilitation strategies used, how well they worked, if lesson goals were met, and how the teaching session can be improved in future [25]. Some examples of reflective questions include [31]:

- What went well during the lesson?
- What can be improved?
- Did the lesson cover the learning objectives I set at the start of the lesson?
- Was my questioning technique effective?
- How well did I engage learners?
- Where can I improve next time?

Gaining feedback from learners can also assist in self-reflective practices [31]. This can be done verbally, by asking “what were the key messages” from the session, and what areas were “confusing” or “least well understood”? Additionally, written feedback may be sought. Having a peer observe facilitation provides another great source of feedback [24]. Tips for receiving feedback include: [26]

- ◦ Be open to the feedback being given as it is intended to be helpful
- ◦ Avoid instantly dismissing feedback that does not match self-reflection
- ◦ Avoid becoming defensive - instead engage in constructive discussion
- ◦ Ask for specific examples to explain the feedback being given.

### Resolving common problems in small group facilitation

Although small group teaching offers many advantages, it may pose some difficulties and limitations for the facilitator and students. Group problems commonly stem from [19, 26, 32]:

- Students being reluctant to engage in discussion with each other
- Students are not prepared for small group activities
- Individual ‘free riders’ failing to contribute (may be shy or disinterested)
- Individual students dominating discussion or being disruptive
- Attention being directed towards the facilitator, who is expected to provide answers
- Facilitator’s questions don’t go beyond the level of recall
- Facilitator’s lack of attempt to get students to answer their own questions
- Facilitators providing insufficient/poor feedback
- Facilitators talking too much, lecturing rather than facilitating.

The facilitator should reflect on why the problem is occurring, what can be done differently to help overcome the problem, and how accountability for success can be shared with the student group [32]. Depending on the specific problem with the group dynamics, sometimes identifying the problem and sharing this perception with the group may prompt the group to solve the problem. However, specific and appropriate strategies may be needed. For example:

*Individual dominant students*: summarise points and divert the discussion to others; indicate time pressure; give the group specific tasks.

*Quiet students:* give them time to respond; divide the group into pairs for a task; positively reinforce any contribution.

*Attention being directed towards the facilitator*: build on students’ responses to a limited extent by sharing clinical experiences or provide a clinical context where appropriate to heighten the relevance of the topic.

*Students receiving insufficient feedback:* schedule a time for student feedback throughout the session, or afterward.

*Students attending unprepared for small group activities*: ensure student accountability to their team members by including short tests at the beginning of class, which may also help to prevent late arrivals [32].

## Conclusion

Small group teaching can be very rewarding, for both the learners and facilitators. However, successful small group facilitation requires appropriate facilitation methods to encourage active and purposeful participation, and enhance student learning. The facilitator’s role is crucial in encouraging the learners to interact with the content, and with their peers. Self-reflective practices and the use of feedback provide a valuable means to improve small group facilitation skills.

### Take-home message

**Table:** 

• Ensure small group activities remain learner-centred, with active participation and purposeful activities.	
• Pay attention to group dynamics to ensure achievement of tasks and effective group work.	
• Use open questions to encourage clinical reasoning, and monitor learners’ progress.	
• Reflect on teaching experience, and gain feedback from participants.	

## Data Availability

Not Applicable.

## Abbreviations

PBL: Problem based learning
TBL: Team-based learning
CBL: Case based learning
